# Insulin receptor substrate-4 interacts with ubiquitin-specific protease 18 to activate the Jak/STAT signaling pathway

**DOI:** 10.18632/oncotarget.22510

**Published:** 2017-11-18

**Authors:** Baihai Jiao, Xuezhen Shi, Yanzhao Chen, Haiyan Ye, Min Yao, Wenxu Hong, Shilin Li, Xiaoqiong Duan, Yujia Li, Yancui Wang, Limin Chen

**Affiliations:** ^1^ Institute of Blood Transfusion, Chinese Academy of Medical Sciences and Peking Union Medical College, Provincial Key Laboratory for Transfusion-Transmitted Infectious Diseases of Sichuan Province, Chengdu 610052, China; ^2^ Key Laboratory of Shenzhen for Histocompatibility and Immunogenetics, Shenzhen Blood Center, Shenzhen 518000, China; ^3^ Toronto General Research Institute, University Network and University of Toronto, Toronto M5G 1L6, Canada

**Keywords:** USP18, IRS4, Jak/STAT signaling pathway, HCV

## Abstract

Ubiquitin-specific protease 18 (USP18) as a negative regulator of the Jak/STAT signaling pathway plays an important role in the host innate immune response. USP18 has been shown to bind to the type I interferon receptor subunit 2 (IFNAR2) to down-regulate the Jak/STAT signaling. In this study, we showed that insulin receptor substrate (IRS)-4 functioned as a novel USP18-binding protein. Co-precipitation assays revealed that two regions (amino acids 335–400 and 1094-1257) of IRS4 were related to bind to the C- terminal region of USP18. IRS4 binding to USP18 diminished the inhibitory effect of USP18 on Jak/STAT signaling. IRS4 over-expression enhanced while IRS4 knock-down suppressed the Jak/STAT signaling in the presence of IFN-a stimulation. As such, IRS4 increased IFN-a-mediated anti-HCV activity. Mechanistically, IRS4 promoted the IFN-a-induced Jak/STAT signaling by interact with USP18. These results suggested that IRS4 binds to USP18 to diminish the blunting effect of USP18 on IFN-a-induced Jak/STAT signaling. Our findings indicated that IRS4 is a novel USP18-binding protein that can be used to boost the host innate immunity to control HCV, and potentially other viruses that are sensitive to IFN-a.

## INTRODUCTION

Interferons (IFN) are broadly divided into three classes: type I IFN, type II IFN, and type III IFN, based on different types of receptors with which they bind [1]. Due to the ability to suppress virus replication and regulate immune systems, type I IFNs (IFN-α and IFN-β) have been used to treat hepatitis B virus, hepatitis C virus infections, autoimmune diseases and several cancers [2–4]. IFN-α and IFN-β interact with their specific receptors (IFNAR1 and IFNAR2), and activate signal transducer and activator of transcription (STAT) and Janus activated kinase (Jak) signaling pathway. As a downstream activation of Jak-STAT signaling pathway, hundreds of interferon stimulated genes (ISGs) are upregulated [5, 6]. Ubiquitin-specific protease 18 (USP18) belongs to the ubiquitin-specific proteases (UBP) family of enzymes [7]. Previous studies demonstrated that USP18 can be induced by viral infections and IFN treatment, suggesting that USP18 may play a critical role in inflammation and host innate immune response [8].

In our previous microarray gene expression profiling study, we identified an 18-gene response signature that differentiated treatment responders from non-responders to IFN treatment of HCV patients [9]. One of these 18 genes is USP18. Increased USP18 expression in the pretreatment liver tissue predicted treatment non-response. Moreover, HCV clearance was associated with more rapid down-regulation of endogenous hepatic USP18 [10]. Studies from ours and others demonstrated that higher expression levels of USP18 inhibited IFN-a anti-HBV and HCV activity in chronic HBV- and HCV-infected patients [11, 12]. Further research from our group indicated that silencing USP18 potentiated IFN anti-HCV activity through activation of the Jak/STAT signaling pathway [13]. Most recently, another study also showed that knockdown of USP18 significantly inhibited HBV replication [11]. These data collectively demonstrated that USP18 played a critical role in IFN resistance.

USP18 is a specific proteinase that cleaves ISG15 from ISG15-conjugated proteins. Several cellular proteins had been identified to interact with USP18, such as IFNAR2 [14], Transforming growth factor beta-activated kinase 1 (TAK1) [15], NF-Kappa-B Essential Modulator (NEMO) [15]. Interestingly, increased USP18 expression is only involved in resistance to IFN-a, but not to IFN-β [16]. Since both IFN-a and IFN-β employed the same receptor IFNAR to activate the Jak/STAT signaling, therefore, it is likely that the difference in resistance to IFN-a and IFN-β is the result of other interacting partners of USP18 other than IFNAR2. Towards this end, we performed immunoprecipitation (IP) and massspectrometry with respect to USP18. Several novel USP18-interacting proteins, among which is insulin receptor substrate-4 (IRS4), were identified. IRS4 belongs to a family of insulin receptor substrate (IRS) which contain IRS1, IRS2 and IRS4 in humans [17]. IRS proteins consist of two highly conserved domains in the N-terminal region, a pleckstrin homology (PH) domain and a phosphotyrosine-binding (PTB) domain, followed by a long, non-conserved C-terminal. IRS family proteins acting as cellular adaptor molecules are important regulatory factors in insulin signaling pathways [18]. Upon insulin stimulation, tyrosine residues of IRS is phosphorylated by the activated insulin receptor, the IRS proteins recruit and activate various adapter molecules or enzymes, such as phosphoinositide 3-kinase(PI3K) and mitogen-activated protein kinase (MAPK) to facilitate glucose uptake [19, 20], lipid metabolism [21] and cell proliferation [22, 23].

In this study, we showed that IRS4 binds to the C-terminus of USP18. Over-expression of IRS4 positively regulates Jak/STAT signaling pathway through diminishing the inhibitory effect of USP18 on IFN signaling and anti-HCV activity. And this IRS4/USP18 interaction diminish USP18′s inhibitory effect on Jak/STAT signaling, therefore to potentiate the anti-HCV effect of IFN-a.

## RESULTS

### Identification of IRS4 as an USP18-interacting Protein

**Figure 1:**
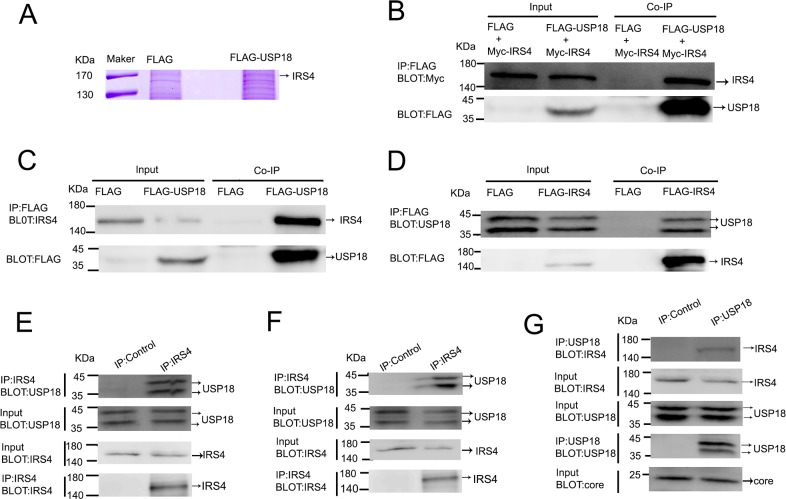
USP18 interacted with IRS4 **(A)** identification of USP18-binding proteins. 293T cell lines stably expressing Flag or Flag-USP18 were established. Cell protein lysates were precipitated with anti-Flag M2 affinity gel. The immunoprecipitated protein complexes were separated by SDS–PAGE, stained using Coomassie R-350 and analyzed by mass spectrometric analysis. **(B)** IRS4 interacted with USP18. 293T cells were co-transfected with Myc-IRS4 and Flag-USP18 or Flag empty plasmid. Cell lysates were immunoprecipitated (IP) with an anti-Flag antibody (incubated with anti-Flag M2 Affinity Gel) and analyzed by immunoblotting with anti-Myc (upper) and anti-Flag antibodies (lower). **(C)** Co-IP assay of the interaction between USP18 and endogenous IRS4. 293T cells were transfected with Flag or Flag-USP18. Cell protein lysates were immunoprecipitated with an anti-Flag antibody followed by immunoblotting with anti-IRS4(upper) and anti-Flag antibodies (lower). **(D)** Co-IP assay of the interaction between IRS4 and endogenous USP18. 293T cells were transfected with Flag or Flag-IRS4. Cell protein lysates were immunoprecipitated with an anti-Flag antibody followed by immunoblotting with anti-USP18(upper) and anti-Flag antibodies (lower). **(E, F)** IRS4 interacts with USP18 endogenously. 293T cells(*E*) and Huh7.5.1 cells(*F*) lysates were immunoprecipitated with an anti-IRS4 antibody or control IgG and analyzed by immunoblotting with anti-USP18 and anti-IRS4 antibodies. **(G)** IRS4 interacts with USP18 endogenously in JFH1-infected Huh7.5.1 cells. JFH1-infected Huh7.5.1 cells lysates were immunoprecipitated with an anti-IRS4 antibody or control IgG and analyzed by immunoblotting with anti-USP18, anti-IRS4 and anti-core antibodies.

The combination of IP and mass spectrometry (MS) was employed to search for cellular proteins that interact with USP18. As shown in Figure 1A, IRS4 was identified as one of the USP18-interacting proteins. The whole band image of staining protein with Coomassie Blue see Supplementary Figure 1. The identified USP18 interaction protein using immunoprecipitation method were listed in Supplementary Table 1. To confirm whether USP18 specifically binds to IRS4, immunoblot analysis of whole cell lysates and anti-FLAG M2 affinity IP derived from 293T cells after cotransfection with plasmids encoding Flag (empty vector) or Flag-tagged USP18 and Myc-tagged IRS4 were performed. Co-IP assay demonstrated that USP18 binds to IRS4 (Figure 1B). To examine whether over-expressed USP18 can interact with endogenous IRS4, Flag or Flag-tagged USP18 were over-expressed in 293T cells and immunoprecipitated with an anti-Flag M2 affinity assay. Co-IP assay clearly demonstrated that USP18 interacts with endogenous IRS4 (Figure 1C). Similarly, over-expressed exogenous IRS4 was also able to interact with endogenous USP18 (Figure 1D). And to detect whether IRS4 interacts with USP18 endogenously, immunoblot analysis of whole cell lysates and anti-IRS4 or lgG IP derived from Huh7.5.1 cells or 293T cells were performed. Our results demonstrated that anti-IRS4 antibody immunoprecipitates endogenous USP18 in both Huh7.5.1 cells (Figure 1F) and 293T cells (Figure 1E). Lastly, to examine whether IRS4 interacts with USP18 endogenously in JFH1-infected cells, immunoblot analysis of whole cell lysates and anti-USP18 or lgG IP derived from JFH1-infected Huh7.5.1 cells were performed. The results demonstrated that anti-USP18 antibody immunoprecipitates endogenous IRS4 in JFH1-infected Huh7.5.1 cells (Figure 1G). These results collectively demonstrated that IRS4 interacted with USP18 endogenously.

### Mapping of USP18-binding region of IRS4

**Figure 2:**
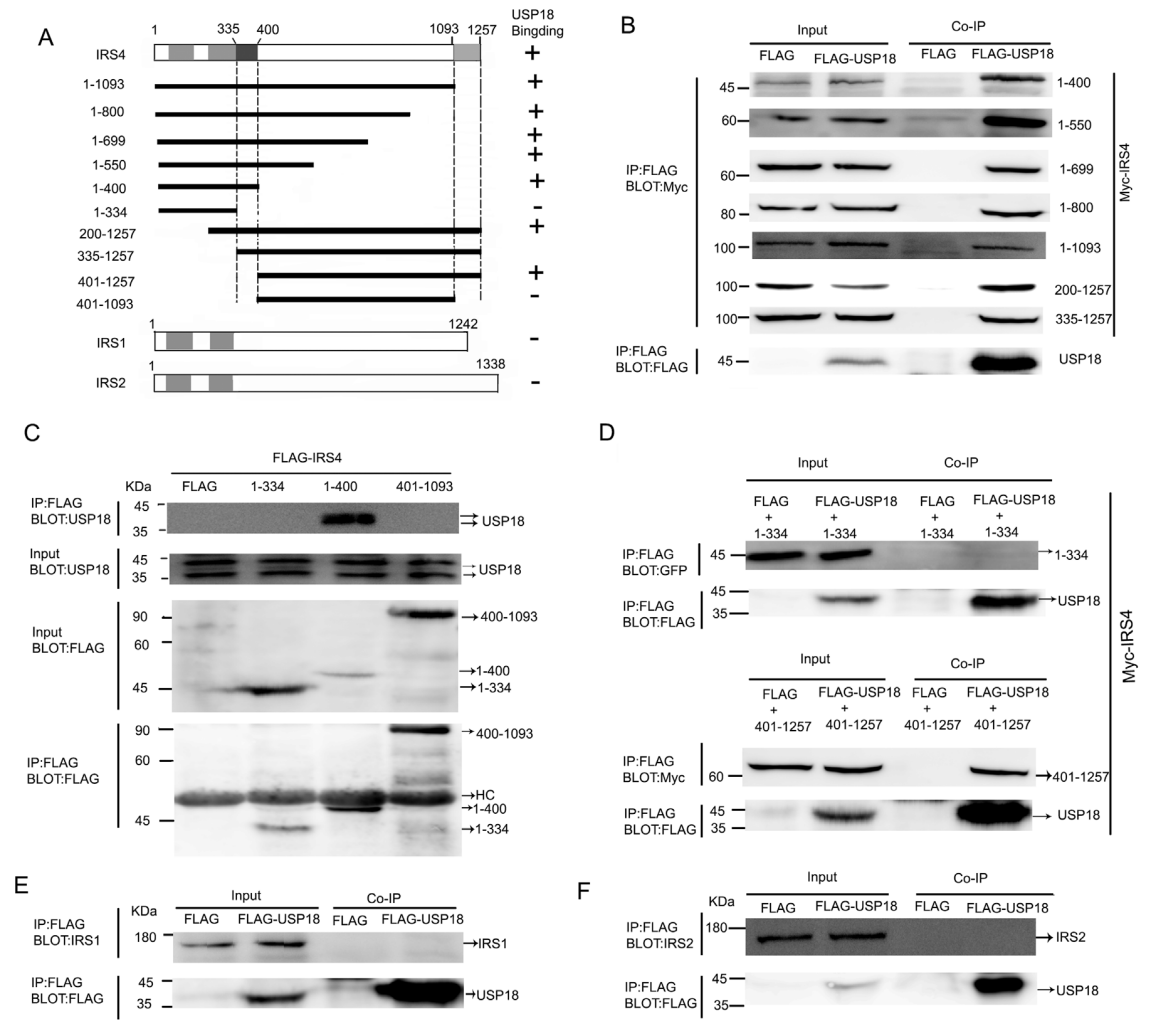
Mapping of USP18-binding regions of IRS4 **(A)** Schematic depiction of human IRS4 and its deletion mutants used in this study. *PH* and *PTB* indicate the pleckstrin homology (PH) domain and phosphotyrosine-binding (PTB) domain, respectively. Summary of the binding domains of IRS4 with USP18 was listed on the right. A major USP18-binding region (amino acids 335–400 and 1094-1257) is indicated. **(B–D)** co-immunoprecipitation assays. (B) 293T cells co-expressing Flag-USP18 and Myc-IRS4 mutant's proteins were lysed and immunoprecipitated with an anti-Flag antibody. Immunoprecipitates (IP) and cell protein lysates (Input) were analyzed by immunoblotting with anti-Myc and anti-Flag antibodies. The figure shows only the bait protein of 1-400. (C) Co-IP assay of the interaction between three IRS4 mutants and endogenous USP18. Cell protein lysates were then immunoprecipitated with an anti-Flag antibody followed by immunoblotting with anti-USP18 and anti-Flag antibodies. HC, heavy chain. (D) 293T cells co-expressing Flag-USP18 and YFP-IRS4 mutant's proteins was lysed and immunoprecipitated with an anti-Flag antibody. Immunoprecipitates (IP) and cell protein lysates (Input) were analyzed by immunoblotting with anti-GFP and anti-Flag antibodies. **(E, F)** Co-IP assay of the interaction between USP18 and endogenous IRS1 and IRS2. Cell lysates were immunoprecipitated with an anti-Flag antibody followed by immunoblotting with anti-IRS1 or -IRS2(upper) and anti-Flag antibodies (lower).

IRS family members include IRS1, IRS2, IRS3 and IRS4. Humans have IRS1, IRS2 and IRS4, while rodents also have IRS3. IRS family members shares a highly conserved pleckstrin homology (PH) domain and a phosphotyrosine-binding (PTB) domain in their N-terminal regions. To identify the USP18-binding region of IRS4, the individual Myc-tagged, Flag-tagged, or YFP-tagged selected regions of IRS4 were co-expressed with Flag-tagged- or His-tagged USP18 in 293T cells (Figure 2A). Co-IP assay demonstrated that IRS4 (1–400) bound to USP18 (Figure 2B). However, IRS4(1–334) did not bind to USP18 (Figure 2D). These findings indicated that IRS4 region 335–400 was a binding region to USP18. Moreover, Co-IP assay of the interaction of Flag-tagged deletion mutants of IRS4 with endogenous USP18 confirmed these results (Figure 2C). In addition, IRS4 (401–1257) bound to USP18 (Figure 2D), but not IRS4 region 401-1093 (Figure 2C). Taken together, the findings indicated that IRS4 335–400 and 1094–1257 regions were related to interact with USP18. Although the IRS family is highly conserved at the N-terminal PH and PTB regions, IRS1 and IRS2 have no consensus sequence to the corresponding IRS4 regions 335–400 and 1094–1257. Therefore, IRS1 and IRS2 did not interact with USP18 (Figure 2E, 2F).

### Mapping of IRS4-binding region of USP18

**Figure 3:**
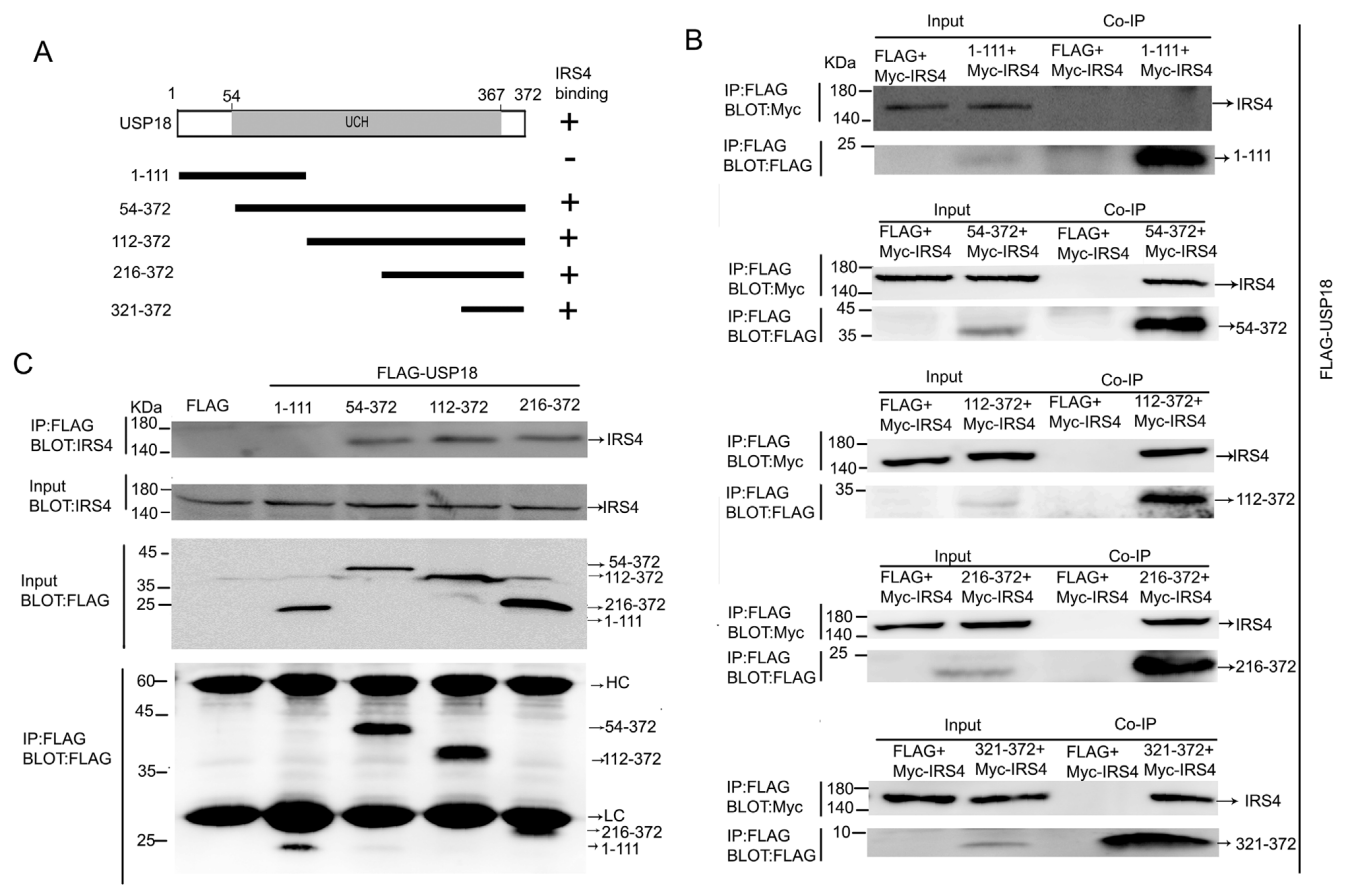
Mapping of IRS4-binding regions of USP18 **(A)** Schematic structure of human USP18 and its deletion mutants used in this study, *UCH* indicate the UCH (ubiquitin C-terminal hydrolase) domains. The IRS4 binding domains is indicated in the *right column*. **(B)** Co-IP assay of the interaction between USP18 mutants and Myc-IRS4. 293T cells co-expressing Flag-USP18 mutants and Myc-IRS4 were lysed and immunoprecipitated with an anti-Flag antibody. Immunoprecipitates and cell lysates were analyzed by immunoblotting with anti-Myc or anti-Flag antibody. **(C)** Co-IP assay of the interaction between USP18 mutants and endogenous IRS4. Cell protein lysates were then immunoprecipitated with an anti-Flag antibody followed by immunoblotting with anti-IRS4(upper) or anti-Flag antibody (lower). HC, heavy chain; LC, light chain.

To determine which region of USP18 binds to IRS4, the Flag-tagged deletion mutants of USP18 was used for binding studies (Figure 3A). The individual Flag-tagged deletion mutants of USP18 and Myc-tagged IRS4 were co-expressed in 293T cells. Immunoblot analysis of whole cell lysates and anti-FLAG M2 affinity IP. Co-IP assay demonstrated that deletion of USP18 N-terminal region (amino acid residue 1-111) did not affect its interaction with IRS4. In contrast, we showed that USP18 C-terminal region (amino acid residue 321-372) bound to IRS4 (Figure 3B). Co-IP assay of the interaction of Flag-tagged deletion mutants of USP18 and endogenous IRS4 confirmed these observations (Figure 3C). The C-terminal region of USP18 (amino acid residue 312–368) has been reported to bind with the type I IFN receptor (IFNAR2) and blocking the Jak1-IFNAR2 interaction leading to the repression of Jak/STAT signaling in mice [14].

### IRS4 enhanced the IFN-a-activated Jak/STAT signaling pathway through interaction with USP18

**Figure 4:**
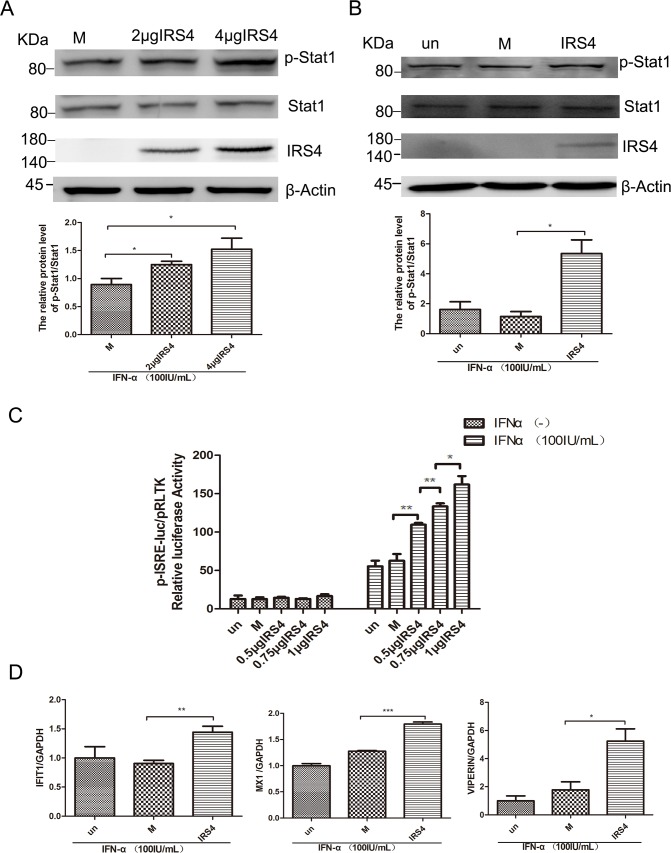
Overexpression of IRS4 enhanced IFN-a-induced activation of Jak/STAT signalling **(A, B)** Overexpression of IRS4 enhanced IFN-a stimulated p-STAT1 level. 293T cells (A) or Huh-7.5.1 cells (B) were transfected with Flag (M, mock control), Flag-IRS4 or untreated(un), the cells were treated with 100 IU/mL IFN-α for 30 mins at 48 hours after transfection. Protein lysates were harvested, separated by SDS-PAGE, and probed for STAT1-phospho701. Blots then were stripped and probed for total STAT1 expression. The ratio of p-STAT1 to total STAT1 from 3 independent experiments was quantified (bottom). **(C)** Overexpression of IRS4 promoted IFN-a-stimulated ISRE activity. Huh7.5.1 cells were co-transfected with Flag-IRS4 plasmid or Flag empty plasmid and pISRE-luc (expressing firefly luciferase) and pRL-TK (expressing Renilla luciferase) as described in Materials and Methods. **(D)** Overexpression of IRS4 promoted IFN-a-induced ISG expression. Expression levels of IFIT1/VIPERIN/MX1 were examined by real time PCR in Huh7.5.1 cells transfected with IRS4 plasmid, empty plasmid (M, mock control) or untreated (un) 24 hours after transfection and then treated with 100IU/mL IFN-α for 24 hours. Error bars indicated mean±SD, ^*^P<0.05, ^**^P<0.01, ^***^P<0.001.

USP18 has been known as a negative regulator of type I IFN signaling. It has been reported that C-terminal region of USP18 (amino acid residue 312–368) competes with Jak1 for interacting with the type I IFN receptor (IFNAR2) and represses downstream Jak/STAT signaling pathway [14]. We also identified C-terminal region of USP18 (321-372) interacted with IRS4 (Figure 3B). We then moved on to explore the effects of IRS4 on the Jak/STAT signaling pathway. Quite interestingly, we found that IRS4 over-expression significantly increased p-STAT1 expression levels in 293T cells (Figure 4A) and in Huh7.5.1cells (Figure 4B) in the presence of IFN-a stimulation. In addition, we found that 100 IU/mL IFN-α induced ISRE activity was further upregulated in the dose dependent manner in the presence of IRS4 over-expression (Figure 4C). In line with this observed increased p-STAT1 expression and ISRE activity, some ISG expression levels were also induced in response to IFN-α stimulation in the presence of IRS4 over-expression (Figure 4D). Furthermore, Two IRS4 mutants (1-334, 401-1093), which did not interact with USP18, did not affect Jak/STAT signaling (Supplementary Figure 2). Taken together, these findings indicated that over-expression of IRS4 enhanced the IFN-a-activated Jak/STAT signaling pathway through interaction with USP18.

### IRS4 knock-down suppressed the IFN-a-induced activation of Jak/STAT signaling

**Figure 5:**
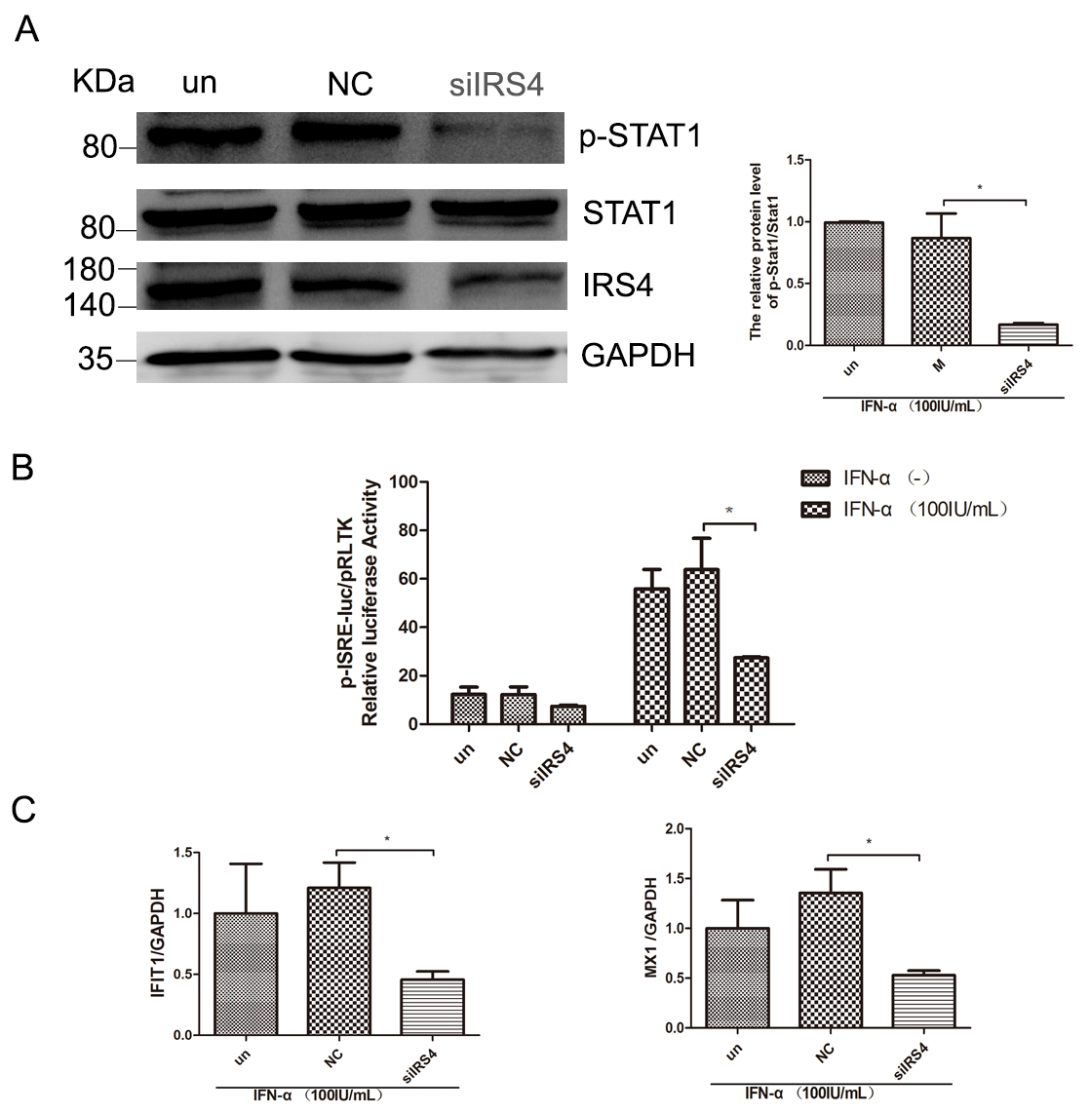
IRS4 knock-down suppressed the IFN-a-induced activation of Jak/STAT signaling **(A)** IRS4 knock-down inhibited IFN stimulated p-STAT1 level. Huh-7.5.1 cells were transfected with siIRS4, negative control (NC) or untreated (un), the cells were treated with 100 IU/mL IFN-α for 30 min at 48 hours after transfection. Protein lysates were harvested, separated electrophoretically, and probed for STAT1-phospho701. Blots then were stripped and probed for total STAT1 expression. The ratio of p-STAT1 to total STAT1 from 3 independent experiments was quantified (right). **(B)** IRS4 knock-down inhibited IFN stimulated ISRE activity. Huh7.5.1 cells were co-transfected siRNA and negative control with pISRE-luc (expressing firefly luciferase) and pRL-TK (expressing Renilla luciferase) as described in Materials and Methods. 24 hours later, cells were treated with 100 IU/mL IFN-α for 24 hours before the cells were lysed for dual luciferase reporter gene assay. **(C)** IRS4 knock-down inhibited IFNa-induced ISG expression. Expression levels of IFIT1/MX1 were examined by real time PCR in Huh7.5.1 cells transfected with siRNA and negative control (NC) or untreated (un), 24 hours after transfection and then treated with 100IU/mL IFN-α for 24 hours. Error bars indicated mean±SD, ^*^P<0.05.

Having confirmed that over-expression of IRS4 enhanced IFN-a-induced activation of Jak/STAT signaling, we then moved on to study the role of IRS4 silencing in this pathway. As expected, IRS4 knockdown caused reduction of p-STAT1 levels in Huh7.5.1 cells in the presence of 100 IU/mL IFN-a stimulation (Figure 5A). In line with this observation, IFN-α induced ISRE activity (Figure 5B) and selected down-stream ISGs expression were also inhibited in Huh7.5.1 cells (Figure 5C). Taken together, these findings indicated that IRS4 knock-down suppressed the IFN-α-induced activation of Jak/STAT signaling.

### IRS4 diminished the inhibitory effects of USP18 on Jak/STAT signaling pathway

**Figure 6:**
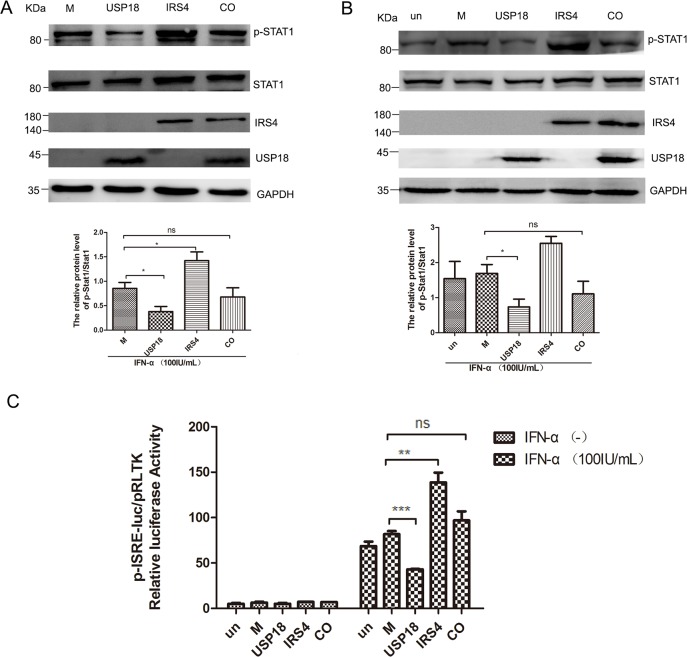
IRS4 promoted IFN-a-induced Jak/STAT signaling pathway through interact with USP18 IRS4 diminished the inhibitory effects of USP18 on Jak/STAT signaling pathway **(A-C)**. 293T cells (A) and Huh7.5.1 cells (B) were transfected with single empty plasmid (M, mock control), Flag-USP18 and Myc-IRS4, or co-transfected with USP18 and IRS4. The cells were treated with 100IU/mL IFN-α for 30 mins at 48 hours after transfection. Protein lysates were harvested, separated by SDS-PAGE, and probed for STAT1-phospho701 and total STAT1 expression. The ratio of p-STAT1 to total STAT1 from 3 independent experiments was quantified (bottom). (C) USP18 expression Flag-tagged plasmid (M, mock control), IRS4 expression Myc-tagged plasmid were co-transfected together with plasmids pISRE-luc and pRL-TK into Huh7.5.1 cells as described in Materials and Methods. 24 hours later, cells were treated with 100IU/mL IFN-α for 24 hours before the cells were lysed for dual luciferase reporter gene assay for ISRE activity. Error bars indicated mean±SD, ^*^P<0.05, ^**^P<0.01, ^***^P<0.001.

To explore whether IRS4 enhanced the IFN-a-induced activation of Jak/STAT signaling pathway through diminishing the inhibitory effect of USP18 on IFN signaling. IRS4 was single- or co-expressed with USP18 in 293T or Huh7.5.1 cells. As expected, exogenous USP18 expression significantly inhibited IFN-a-induced Jak/STAT signaling as shown by decreased p-STAT1 levels (Figure 6A, 6B) and ISRE activity (Figure 6C). The p-STAT1 expression level (Figure 6A) in 293T cells and Huh7.5.1 cells (Figure 6B) and the ISRE activity (Figure 6C) were restored in the IRS4/USP18 co-expressed group. These results collectively demonstrated that IRS4 diminished the inhibitory effect of USP18 on Jak/STAT signaling pathway.

### IRS4 regulated HCV replication in JFH1 infected Huh7.5.1 cells

**Figure 7:**
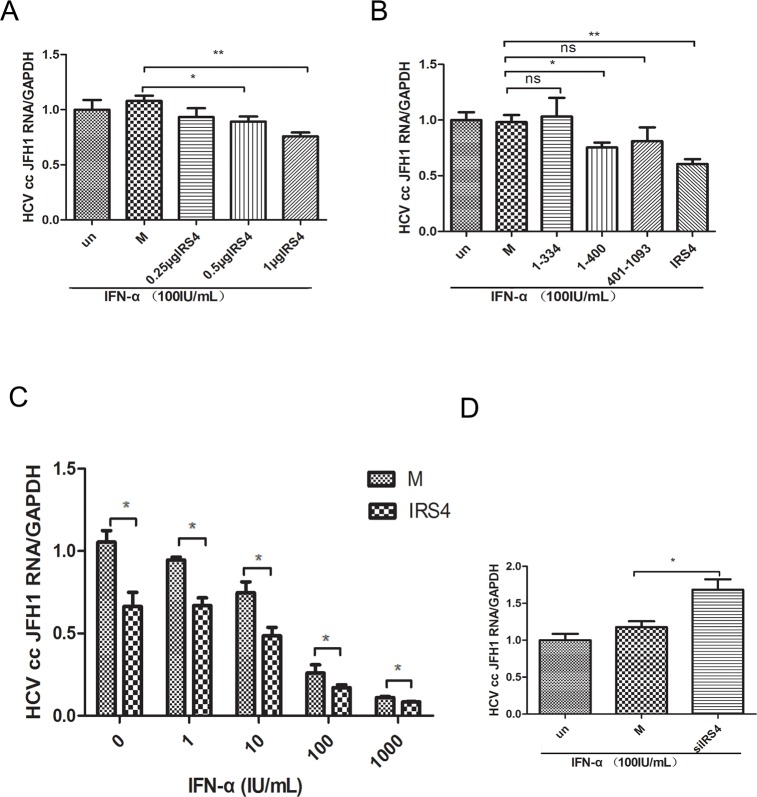
IRS4 regulated HCV replication in JFH1 infected Huh7.5.1 cells Different doses of IRS4 plasmid, Flag-tag empty plasmid (M, mock control) or nothing (un) were transfected into Huh7.5.1 cells infected with HCV JFH1. 24 hours later the cells were treated with 100IU/mL IFN-α **(A)** for 24 hours and the relative expression levels of HCV RNA were examined by real time PCR. **(B)** Two mutants IRS4 (1-334) and IRS4 (401–1093) that do not bind to USP18 have no effect on interferon anti-HCV activity. Flag empty vector (M, mock control) or IRS4 mutant 1-334 or 401-1093 were transfected into Huh7.5.1 cells infected with HCV JFH1. 24 hours later the cells were treated with 100 IU/mL IFN-α (A) for 24 hours and the relative expression levels of HCV RNA were examined by real time PCR. **(C)** Dosage effects of IFN-α (0, 1, 10, 100, 1000 IU/mL) on HCV replication in IRS4-overexpressed Huh7.5.1 cells infected with HCV JFH1. Relative expression levels of HCV RNA were examined by real time PCR. The values are displayed as the expression level of JFH1 HCV relative to Flag-tag empty vector (mock). Error bars indicated mean±SD, ^*^P<0.05, ^**^P<0.01. **(D)** IRS4 knock-down inhibited IFN-α induced anti-HCV activity. JFH1 infected Huh7.5.1 cells were transfected with siIRS4, negative control (NC) or untreated (un), 24 hours later the cells were treated with 100 IU/mL IFN-α for 24 hours and the relative expression levels of HCV RNA were examined by real time PCR.

PEG-IFN is one of the efficient agents approved for the treatment of chronic HCV. We evaluated whether IRS4 enhances IFN-α induced antiviral activity in HCV JFH1 infected Huh7.5.1 (as previously described [24, 25]). After different doses of IRS4 plasmid transfection, the JFH1 infected cells were treated with 100 IU/mL IFN-α for another 24 hours and HCV JFH1 RNA was analyzed by quantitative real-time PCR. We found that IRS4 suppressed HCV RNA replication in dose-response manner in the presence of IFN-α (Figure 7A). Furthermore, two mutants IRS4(1-334) and IRS4(401–1093) that do not bind to USP18 have no effect on interferon anti-HCV activity (Figure 7B). In addition, IRS4-transfected Huh7.5.1 cells infected by JFH1 were treated with indicated dose of IFN-α and cultured for 24 hours, and then HCV JFH1 RNA was quantified. In the presence of IRS4, IFN inhibited HCV RNA replication by 20%-40% (Figure 7C). Lastly, we evaluated whether knockdown IRS4 inhibited IFN-α induced antiviral activity in HCV-JFH1 cell culture system. Huh7.5.1 cells were infected with HCV JFH1 viruses (0.3 MOI) for 4 hours followed by siIRS4 transfection. 24 hours later the cells were treated with 100 IU/mL IFN-α for another 24 hours and HCV JFH1 RNA was analyzed by quantitative real-time PCR. We found that in the presence of IFN-α, silencing IRS4 promote HCV RNA replication by 30%. (Figure 7D). These results suggested that IRS4 potentiated IFN-α antiviral effect against HCV replication through interaction with USP18.

## DISCUSSION

USP18 expression was up-regulated in the pretreatment liver tissues of patients chronically infected with HBV and HCV who do not respond to subsequent treatment with pegylated IFN-a and ribavirin [9, 12] or in patients with DAA therapy [10]. HCV clearance was also associated with more rapid down-regulation of endogenous hepatic ISGs. Studies from our group and others clearly demonstrated that USP18 play a critical role in host innate immune response against HBV and HCV infections [11, 26, 27].

USP18 shares catalytic domains with ubiquitin-binding proteins (UBPs). In human, a mutation of the USP18 within the Cys box at position 64 completely abolishes the protease activity by substituting a single amino acid (Cys◊Ser C64S) [15]. Another USP18 functional domain locates at its C terminus. This domain facilitates USP18 binding to the intracellular domain of the IFNAR2 subunit leading to the suppression of interferon induced Jak/STAT signaling. USP18 binds to IFNAR2 by competing with Jak1, thereby limits the activity of STATs and suppresses IFN response. Therefore, knockdown USP18 leads to prolonged and enhanced the activity of STAT1, and upregulated expression of many ISGs. [28]. Perhaps as a result of this increased IFN signaling and effect, USP18 knock out mice show greater resistance to the cytopathic effects of a number of viruses, including lymphocytic choriomeningitis virus (LCMV), vesicular stomatitis virus (VSV), and Sindbis virus (SNV) [29]. Thus, USP18 has been recognized as a negative regulator of IFN signaling.

Since IFN-α and IFN-β are widely used to treat hepatitis B virus and hepatitis C virus infections [30], USP18 inhibitors may be an effective strategy for modulating IFN antiviral activity. We recently demonstrated that increased expression of USP18 is associated with persistent infection of HCV and viral tolerance to interferon (manuscript in preparation). In contrast, silencing USP18 activates the Jak/STAT signaling and potentiates IFN anti-HCV ativity [13]. We also found silencing USP18 upregulated some ISG expression levels with reduced HBV DNA level [11]. Interestingly, ISGylation is not associated with the replication of HBV [31]. Taken these results together, reduced expression of USP18 is beneficial to the clearance of HBV and HCV. This may be due to the fact that decreased expression of USP18 increases the sensitivity of IFN. In other words, inhibition of USP18 function leads to a strengthened immune response.

In this study, combination of IP and MS screening and *in vitro* functional studies identified that IRS4 interacts with USP18 endogenously. We also showed that USP18 binds to IRS4 primarily through the C-terminal region (amino acids 321-372) and thus to inhibit downstream Jak/STAT signal transduction. We identified that amino acids 335–400 and amino acids 1094-1257 of IRS4 are important for the IRS4-USP18 interaction. Interestingly, these two IRS4 regions are also required for binding to Slingshot-1 (SSH1) [32]. These two regions contributed to the selective interaction of IRS4-USP18 and IRS4-SSH1L. These findings indicated that these two regions were the major interaction sites of IRS4. Although IRS family proteins are essential for modulate insulin signaling pathway [33–36], we also found overexpression of IRS4 significantly promoted while IRS4 knock-down suppressed the IFN-α-induced activation of Jak/STAT signaling. In this study, we used JFH1 HCV culture model to dissect the role of IRS4/USP18 interaction in IFN-a anti-HCV activity. We found that overexpression of IRS4 significantly reduced while knockdown IRS4 promoted the intracellular replication level of HCV RNA in the presence of IFN-a in JFH1-infected cells. These findings suggested that IRS4 enhanced the antiviral of IFN-α against HCV replication. Although IRS4 has been shown to be involved insulin signaling pathways and PI3-Kinase signaling [17, 37], we show that IRS4 is also associated with interferon induced activaiton of Jak/STAT signaling pathway. Many research showed that HCV infection can induce insulin resistance (IR) in the liver through multiple mechanisms which interferes with insulin signaling pathway both directly and indirectly, inducing the production of several proinflammatory cytokines [38–40]. IR has been documented in patients with chronic HCV, playing a critical role in the progression of hepatic fibrosis, cirrhosis and hepatocellular carcinoma. But the underlying mechanism of this association is not clear. Takumi'et al [38] showed that HCV core protein downregulated IRS1 and IRS2 though ubiquitination by SOCS3. This indicated that IRS1 and IRS2 were related to HCV-associated insulin resistance. But it is unknown whether IRS4 are related to HCV-associated insulin resistance. And precious studies showed that SOCS −1, −3, −6 and −7 disrupt insulin signaling through interacting insulin receptor IRS1 and IRS2 [39]. And in this study, we found that IRS4 functioned as a novel USP18-binding protein, but IRS1 and IRS2 do not bind to USP18. Therefore, it is likely that USP18 modify insulin signaling through a unique mechanism compared with SOCS −1, −3, −6 and −7. Maybe the unique mechanism is the interaction with USP18 and IRS4.

**Figure 8:**
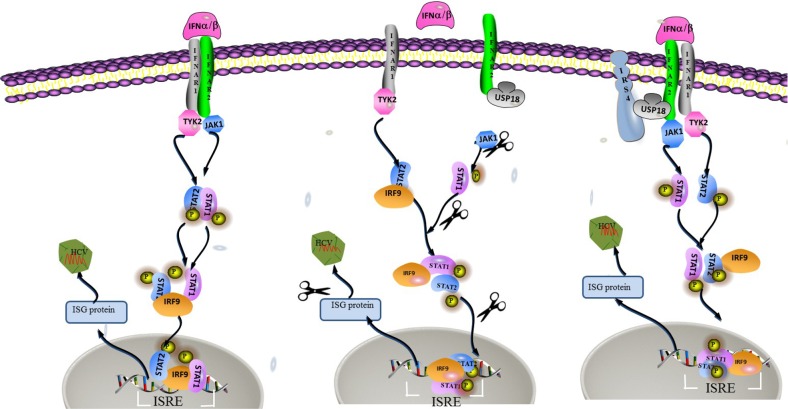
A hypothetical model of IRS4/USP18 interaction and its role in IFN antiviral function USP18 binding to IFNAR2 *in vivo* blocks the interaction between Jak and the IFN receptor, thereby reduces the phosphorylation of the receptor and STATs and suppresses signal pathway. The interaction between IRS4 and USP18 decreased the inhibitory effect of USP18 on Jak/STAT signaling pathway. Therefore, IRS4 enhanced IFN-α induced activation of the Jak/STAT signaling pathway as shown by the increased levels of p-STAT1 and enhanced ISRE activity and increased induction of ISGs.

Our results suggested that overexpressed IRS4 promoted while knockdown IRS4 inhibited the Jak/STAT signaling pathway by the interaction of IRS4 and USP18. And IRS4 regulated HCV infection through enhanced activation of the Jak/STAT signaling pathway by counteracting the inhibitory effect of USP18. A schematic depiction of IRS4-USP18 regulation of the IFN response is shown in Figure 8.

## MATERIALS AND METHODS

### Cells, cell culture and HCV JFH-1 virus

Huh7.5.1 cells were kindly provided by Professor Zhongtian Qi from the Second Military Medical University (Shanghai, China). HCV JFH-1 cell culture system (HCV genotype 2a) was provided by Dr. Charles Rice from Rockefeller University. Huh7.5.1 cells were maintained in Dulbecco's modified Eagle's medium (DMEM; Thermo) with 10% (v/v) fetal bovine serum (FBS; GIBCO), 1% Penicillin-Streptomycin (P/S) (Hyclone, Toronto, Canada) and 1% non-essential amino acid (Thermo). 293T cells were maintained in DMEM (Hyclone) with 10% fetal bovine serum (Gibco) and Penicillin-Streptomycin (P/S) (Hyclone, Toronto, Canada) at 37°C in 5% CO_2_ incubator.

### Plasmid construction

Plasmids expressing USP18, IRS4 were constructed with routine molecular cloning techniques. The full length human USP18 gene was amplified by polymerase chain reaction (PCR) from total RNA isolated from Huh7 cells and cloned into pcDNA3.1-3^*^tag (Flag, His and StrepII) or pDest26 (Invitrogen) to create the mammalian expression constructs pcDNA3.1-USP18 or pDest26-USP18. Full length and deletion mutants of IRS4 were subcloned into pEYFP-C1 (Clontech) or FPC1-Myc vector which was kindly provided by Dr. Kensaku Mizuno from Tohoku University, Japan [32]. Full length and some mutants of IRS4 (1-334, 1-400, 401-1093) were also subcloned into p3XFLAG-CMV-7.1(SIGMA). All the constructs were sequence-verified.

### IRS4 knockdown

siRNA targeting human IRS4 (Sense 5′ GGCCUAGACAAAGAAGUCUTT3′; Antisense 5′AGACUUCUUUGUCUAGGCCTT3′), negative control siRNA (Sense 5′ UUCUCCGAACGUGUCACGUTT3′ ; Antisense5′ ACGUGACACGUUCGG AGA ATT3′) (Sangon Biotech, China), and Transfection Reagent (Lipofectamine® RNAiMAX) were purchased from Invitrogen. To test the effect of IRS4 silencing on Jak/STAT pathway in the presence of IFN-a stimulation, Huh7.5.1 cells were plated in 6-well plates overnight. Media were changed, and the cells were transfected in solution with siRNA at a final concentration of 50nM according to the manufacturer's protocol. 48 hours later, the culture medium was removed and the cells were washed twice with PBS before IFN-α (final concentration 100IU/mL) was added. 30mins later the cells were harvested and the total proteins were extracted for p-STAT1 analysis (Western Blot). To examine the effect of silencing IRS4 on ISRE activity, Huh7.5.1 cells were seeded in 24-well plates for 24 hours and then transfected with siIRS4. 24 hours later, 0.5μg pRL-TK and 0.5μg pISRE-luc per well was transfected into the cells. 24 hours later, the culture medium was removed and the cells were washed twice with PBS before IFN-α (final concentration 100 IU/mL) was added. Dual-luciferase reporter assay kit (Promega, USA) was used to check the ISRE activity 24 hours later following manufacturer's protocol. To study the effect of silencing IRS4 on ISGs, Huh7.5.1 cells were seeded in 24-well plates for 24 hours and then transfected with siIRS4, 24 hours after transfection, cells were treated with 100 IU/mL IFN-α for another 24 hours and then harvested for mRNA analysis of various ISGs (RT-PCR).

### Immunoprecipitation and mass spectrometry analysis

293T cells were harvested 48h after pcDNA3.1 plasmid or pcDNA3.1-USP18 plasmid transfection. The cells were washed with PBS and lysed in RIPA lysis buffer (Beyotime, China) containing proteinase inhibitor PMSF and centrifuged at 12,000g for 10 min at 4°C. 3mg of lysed protein supernatants were incubated with 20 μL anti-Flag M2 affinity gel (Sigma) overnight at 4°C. The protein-IP mixtures were washed with RIPA lysis buffer and separated by 12% sodium dodecyl sulphate–polyacrylamide gel electrophoresis (SDS–PAGE) and stained with Coomassie R-350. Differentially pull-down proteins were excised from the gel and identified using an in-gel digestion method and matrix-assisted laser desorption/ionization-time of flight mass spectrometry (MALDI-TOFMS) as described previously [41].

### Co-Immunoprecipitation (Co-IP) assay

293T cells or Huh7.5.1 were harvested 48h after co-transfection with indicated plasmids. The harvested protein supernatants were incubated either with 20μL anti-Flag M2 affinity gel (Sigma) or 20μL Protein A&G Agarose (Santa Cruz Biotechnology) with 2μg IRS4 antibody added. After washing with RIPA lysis buffer, the immunoprecipitated complexes were separated by SDS-PAGE and analyzed by western blotting using an appropriate antibody, including anti-Flag monoclonal anti-body (1:1,000 dilution, Sigma), anti-USP18 antibody (1:1,000 dilution; Cell Signaling Technology), anti-IRS4 antibody (1:500 dilution; BBI Life Science Corporation), anti-GFP antibody (1:500 dilution; BBI Life Science Corporation), anti-Myc antibody (1:500 dilution; BBI Life Science Corporation) and anti-HCV core antibody (1:1000; Bioss).

### ISRE-luciferase reporter assay

To study the effect of IRS4 on IFN-α induced ISRE activity, a dual-luciferase reporter assay was performed. Huh7.5.1 cells were seeded at 2.5×10^5^ cells/ mL for 0.5mL each well in 24-well plates for 24 hours before the cells were co-transfected with different doses of IRS4 plasmid DNA together with 0.5μg pRL-TK, and 0.5μg pISRE-luc. The empty vector p3XFLAG-CMV-7.1 served as negative control. And to verify whether IRS4 promoted ISRE activity through the interaction with USP18, Huh7.5.1 cells were co-transfected with 1μg pcDNA3.1-USP18, 1μg p3XFLAG-CMV-7.1-IRS4, 0.5μg pRL-TK, and 0.5μg pISRE-luc. The empty vector pCDNA3.1 and p3XFLAG-CMV-7.1 served as negative controls. 24 hours after transfection, cells were treated with 100 IU/mL IFN-α for another 24 hours and then detected by dual-luciferase reporter assay kit (Promega, USA) following manufacturer's protocol. Each transfection experiment was performed in duplicate and repeated three times.

### Plasmids transfection and quantifcation of HCV production

All the cells were seeded at 2.5×10^5^ cells/ mL for 0.5 mL each well in 24-well plate and 2ml each well in 6-well plate. The ratio of plasmid to transfection reagent (PEI, polyethylenimine) was 1:3. To study the effect of IRS4 on HCV replication and anti-HCV activity of IFN-α, Huh7.5.1 cells were seeded in 24-well plates for 24 hours and then infected with 100μL HCV JFH-1 virus stock (MOI= 0.3) for 4 hours. The culture medium was removed and the cells were washed twice with PBS (Sangon Biotech, Shanghai, China) before transfected with different doses of p3XFLAG-CMV-7.1-IRS4 plasmid and p3XFLAG-CMV-7.1(NC, negative control). 24 hours later, the culture medium was removed and the cells were washed twice with PBS before different amount of IFN-α was added to the final concentration of 1-1000 IU/mL. The cellular total RNA was extracted by TRIzol (Invitrogen, USA) following manufacturer's instructions 24 hours after IFN-α treatment. Real time PCR was performed using HCV or other gene specific primers. The primer sequences were listed in Supplementary Table 2.

### Statistical analysis

Data analysis was performed using a 2-tailed Student's *t*-test. All the data are expressed as mean ± SD of at least three independent experiments. In all analyses, ^*^P < 0.05, ^**^P <0.01, and ^***^P <0.001 for comparison of indicated treatments.

## SUPPLEMENTARY MATERIALS FIGURES AND TABLES


