# Supplementary material for: Insulin receptor substrate-4 interacts with ubiquitin-specific protease 18 to activate the Jak/STAT signaling pathway
Source: Oncotarget. 2017 Nov 18;8(62):105923–35. doi: 10.18632/oncotarget.22510 (PMC5739690; doi:10.18632/oncotarget.22510)
Supplement: Supplementary file 1 [file oncotarget-08-105923-s001.pdf]

Insulin receptor substrate-4 interacts with ubiquitin-specific protease 18 to activate the Jak/STAT signaling pathway

SUPPLEMENTARY MATERIAL

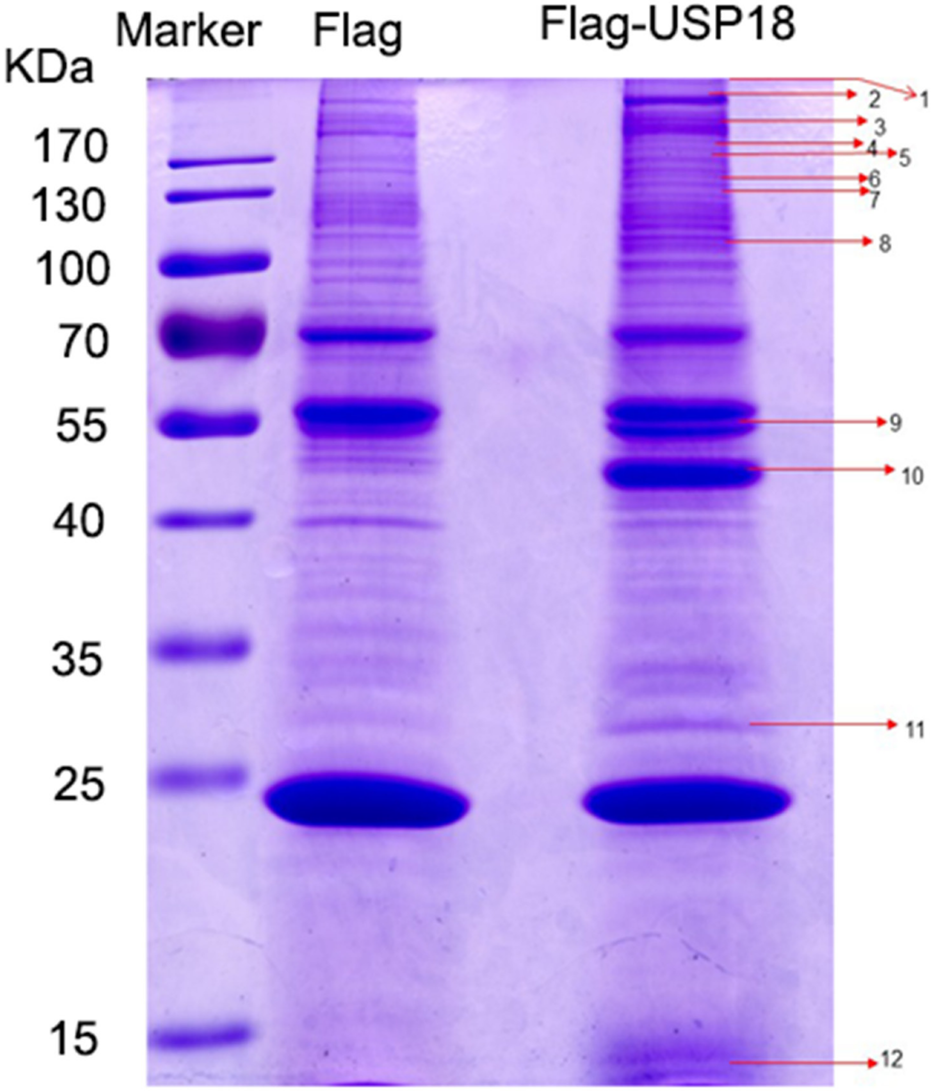

Supplementary Figure 1: Identification of USP18-binding proteins. 293T cell lines stably expressing Flag or Flag-USP18 were established. Cell protein lysates were precipitated with anti-Flag M2 affinity gel. The immunoprecipitated protein complexes were separated by SDS–PAGE, stained using Coomassie R-350 and analyzed by mass spectrometric analysis.

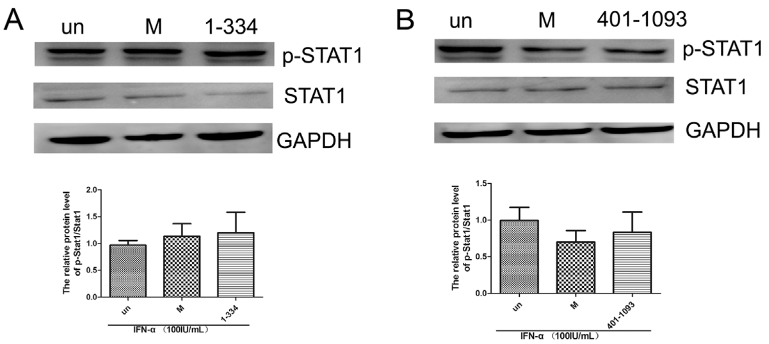

Supplementary Figure 2: IRS4 mutants (1-334, 401-1093) did not affect IFN-a-induced activation of Jak/STAT signaling pathway. Huh-7.5.1 cells were transfected with Flag empty vector or IRS4 mutant1-334 **(A)** or 401-1093 **(B)**, the cells were treated with 100 IU/mL IFN-α for 30 min at 48 hours after transfection. Protein lysates were harvested, separated by SDS-PAGE, and probed for STAT1-phospho701. Blots were then stripped and probed for total STAT1 expression. The ratio of p-STAT1 to total STAT1 from 3 independent experiments was quantified (bottom).

Supplementary Table 1: Identify USP18 interaction protein using immunoprecipitation method

E3 ubiquitin protein ligase HUWE1
DNA dependent protein kinase catalytic subunit
Fatty acid synthase
Ubiquitin-40S ribosomal protein S27a
Insulin receptor substrate 4
ATP-dependent RNA helicase A
Elongation factor 2
Tubulin beta chain
Elongation factor 1-alpha 1
Elongation factor 1-alpha 2
Histone H2B
Histone H4

Supplementary Table 2: Real time PCR primer

Gene name	Primer sequence
GAPDH	Forward 5′-GCCTCCTGCACCACCAACTG-3′
	Reverse 5′-ACGCCTGCTTCACCACCTTC-3′
JFH-1	Forward 5′-GCAGAAAGCGCCTAGCCAT-3′
	Reverse 5′-CTCGCAAGCGCCCTATCAG-3′
Mx1	Forward 5′-GTGCATTGCAGAAGGTCAGA-3′
	Reverse 5′-CTGGTGATAGGCCATCAGGT-3′
IFIT1	Forward 5′- TCAGGTCAAGGATAGTCTGGAG-3′
	Reverse 5′- AGGTTGTGTATTCCCACACTGTA-3′
VIPERIN	Forward 5′-TCTTGAGTGTGTTCAGGCA-3′
	Reverse 5′-TCTTCCTCCTCTTTGGTCTC-3′
